# Comparison of clinical outcomes following periapical surgery in endodontics versus extraction with implant placement

**DOI:** 10.6026/973206300222179

**Published:** 2026-04-30

**Authors:** Arpita Anand, Nitu Chaubey, Jyoti Kumari, Vinayak Singh, Peeyush Kumar Chaudhary, Anmol Mehrotra

**Affiliations:** 1 Department of Conservative Dentistry and Endodontics, Dental College Azamgarh, Azamgarh, Uttar Pradesh, India; 2 Department of Conservative Dentistry and Endodontics, Purvanchal Institute of Dental Sciences, GIDA, Gorakhpur, Uttar Pradesh, India; 3 Department of Conservative Dentistry and Endodontics, KD Dental College and Hospital, Mathura, Uttar Pradesh, India; 4 Department of Conservative Dentistry and Endodontics, Institute of Dental Sciences, Bareilly, Uttar Pradesh, India

**Keywords:** Periapical surgery, apicoectomy, dental implants, clinical outcomes, endodontic treatment, tooth preservation

## Abstract

Persistent periapical lesions challenge clinicians between periapical surgery and extraction-implant, yet long-term comparative outcomes remain limited. Hence, this retrospective cohort followed 166 patients (78 per group) for ≥24 months, comparing success rates, clinical/ radiographic outcomes, satisfaction, cost and duration after failed endodontics. Periapical surgery achieved 89.7% success, compared with 92.3% for implants (p=0.571), with comparable survival, satisfaction and periapical healing; surgery excelled in shorter duration with lower cost. Both modalities yield reliable long-term results without significant differences in efficacy. Thus, patient and tooth-specific factors are needed over modality superiority in decision-making.

## Background:

Natural dentition is one of the cornerstones of modern dental practice. Still, there are also many clinical situations in which a tooth with persistent periapical pathology requires more effective treatment than root canal therapy [1]. In case of failure of initial endodontic treatment to address periapical disease, the process of treatment decision-making is more complex. It demands careful consideration of available therapeutic options, such as non-surgical retreatment, periapical surgery or extraction followed by prosthetic replacement [2]. Periapical surgery, including apicoectomy with retrograde filling, has advanced significantly over the past several decades due to microsurgical techniques, ultrasonic equipment and biocompatible root-end filling materials such as mineral trioxide aggregate [3]. Modern periapical microsurgery incorporates high-power magnification and light that allow accurate definition of anatomical complications such as isthmuses, lateral canals and root fractures, which can be the cause of recurrent illness [4]. Modern periapical surgery has been reported to achieve success rates of 85%-96%, a significant improvement over traditional surgery without a surgical microscope [5]. On the other hand, the development of dental implant technology has made the concept of extractions followed by subsequent implant placements a more reliable treatment option for teeth with a poor long-term prognosis [6]. Implant-supported restorations have been shown to have a survival rate of more than 95% at 10-year follow-ups, offering patients functional and esthetic results similar to those of natural teeth [7]. The introduction of instant implant placement guidelines has also improved treatment effectiveness, as it has shortened the treatment process and the number of surgical procedures [8]. The choice between periapical surgery to preserve teeth and extractions, with replacement of missing teeth with implants, involves several factors, including expected success rates, treatment complexity, recovery time, costs and patient preferences [9]. Some recent studies have attempted to compare these treatment modalities, but the mixed study designs, varying follow-up periods and different outcome measures have made direct comparisons difficult [10].

The evidence from a systematic review of the literature showed that both treatments have high success rates, but prospective comparative studies with standardized assessment criteria remain quite limited [11]. The biological and functional benefits of biological tooth structure conservation are the retention of proprioceptive feedback systems, preservation of periodontal attachment apparatus and alveolar bone volume conservation [12]. Nevertheless, supporters of extraction and placement of the implants point out the eradication of potential sources of chronic infection and the establishment of a reliable base for prostheses that are not influenced by prior endodontic issues [13]. This discussion of stereotypes in the dental field has made it apparent that evidence-based comparative analyses must guide clinical decision- making. Although this is a treatment decision of clinical importance, additional research gaps remain, including a lack of direct outcome comparisons between periapical surgeries and implant placement in similar patient groups [14]. Past research has not been able to account for confounding factors such as tooth position, lesion size and patients' systemic factors that might affect treatment outcomes, regardless of the type of intervention used [15]. Moreover, patient-related outcome indicators such as patient satisfaction, quality of life and treatment experience have not been properly assessed in available comparative research. Therefore, it is of interest to compare the clinical outcomes, radiographic healing, complications and patient satisfaction of periapical surgery and extraction with implant placement in patients with persistent periapical pathology after primary root canal treatment, using standardized assessment criteria and long follow-up to provide complete comparative outcome data.

## Materials and Methods:

## Study design and setting:

The study was a retrospective cohort study conducted in the Department of Oral and Maxillofacial Surgery and the Department of Endodontics at a university dental hospital from January 2018 to December 2022.

## Sample size calculation:

The G*Power software (version 3.1.9.7) was used to analyze power with the following parameters: effect size = 0.45, alpha error probability = 0.05 and power = 0.80. The minimum sample size was estimated at 68 subjects per group. A target of 78 subjects per group was set, accounting for potential dropout and missing records.

## Patient selection:

Patients who met the inclusion criteria were consecutively recruited into one of the two groups: Group A (periapical surgery) and Group B (extraction and implant placement). Treatment allocation was made based on clinical decision-making at the time of presentation, taking into account patient preferences, anatomical factors and restorative prognosis.

## Inclusion criteria:

[1] Age between 18 and 70 years

[2] Radiographically confirmed presence of periapical pathology and lesion diameter 5 mm and above.

[3] Past root canal treatment and recurrent periapical lesion.

[4] A minimum of 24 months follow-up.

[5] Full clinical and radiographic records present.

## Exclusion criteria:

[1] Systemic disease of bone metabolism (uncontrolled diabetes, osteoporosis with treatment with bisphosphonates)

[2] Present tobacco consumption of more than 10 cigarettes per day.

[3] Vertical root fracture preoperatively.

[4] Pregnancy or lactation

[5] Grade III mobility of teeth.

[6] Past periapical surgeries on the tooth.

[7] Poor bone volume to receive implants without augmentation surgeries.

## Surgical procedures:

## Periapical surgery (Group A):

Two qualified endodontic surgeons had a minimum of 10 years of experience in using standardized protocols of microsurgical procedures in all the periapical surgeries. A complete mucoperiosteal flap was elevated after the administration of local anesthesia (4% articaine with 1: 100,000 epinephrine). The osteotomy was done with round surgical burs with a lot of saline running to provide sufficient access to the root apex. Root-end resection (3 mm) was done at right angles to the long axis of the root with a straight fissure bur to a depth of 3 mm and retrograde cavity preparation was done with ultrasonic tips to a depth of 3 mm and root-end filling was done with mineral trioxide aggregate (ProRoot MTA, Dentsply Sirona). Monofilament sutures, 4-0 or 5-0, were then used to reposition the flaps. All procedures were performed under an operating microscope with a magnification of 10-25xs.

## Extractions and implantation (Group B):

Extractions were performed atraumatically, with fewer traumas to the surrounding alveolar structures via periotomes and forceps. The placement of the implant was performed either immediately (within 24 hours of the extraction) or delayed (3-6 months after the extraction), based on clinical evaluation of socket stability and bone supply. The correct size of the titanium implant was fitted as per the manufacturer's instructions and primary stability was assessed using insertion torque values. Bone grafting was done where necessary to counter deficiencies. The restoration of implants was done after 3-6 months with screw-retained or cement-retained single crowns.

## Outcome assessment:s

Clinical and radiographic assessments were conducted at baseline, 3, 6, 12 and 24 months after treatment by two blinded examiners who were unaware of the treatment group assignment. The inter-examiner frequency was determined by using the Cohen kappa coefficient (= 0.87).

## Primary outcome measures:

[1] Success rate: No clinical signs and symptoms (pain, swelling, sinus tract) and radiographic findings of a full recovery or a significant decrease of periapical radiolucency (>50% decrease)

[2] Survival rate: Remained use of the treated tooth or implant notwithstanding clinical or radiographic condition.

## Secondary outcome measures:

Periapical Index (PAI) of periapical healing classification.

[1] The rate of complications (infection, paresthesia, wound dehiscence)

[2] Patient satisfaction measured with a 10-point visual analog scale that has been previously tested and validated.

[3] Time of treatment period and the number of visits necessary.

## Statistical analysis:

SPSS (version 26.0, IBM Corporation) was used to analyze the data. Continuous variables were presented as mean and standard deviation and compared between groups using independent samples t-tests or Mann-Whitney U tests, depending on the test of normality (Shapiro-Wilk). Frequencies and percentages were used to present categorical variables and to compare them using chi-square or Fisher's exact tests, depending on the situation. The Kaplan-Meier method was used for survival analysis, with log-rank tests to compare the groups. All analyses were done at p < 0.05.

## Results:

A total of 156 patients were included in the final analysis, with 78 patients in each treatment group. The mean age was 47.3 ± 12.8 years in Group A and 49.1 ± 11.6 years in Group B. Demographic characteristics and baseline parameters were comparable between groups with no statistically significant differences observed (Table 1 - see PDF). At the 24-month follow-up evaluation, the success rate was 89.7% (70/78) in the periapical surgery group and 92.3% (72/78) in the extraction-and-implant group. This difference was not statistically significant (p = 0.571). The survival rate was 94.9% (74/78) for Group A and 97.4% (76/78) for Group B (p = 0.407). Kaplan- Meier survival analysis demonstrated comparable survival curves between groups (log-rank p = 0.382). Detailed outcome measures are presented in Table 2 (see PDF). The overall complication rate was 14.1% (11/78) in Group A and 11.5% (9/78) in Group B (p = 0.628). In the periapical surgery group, complications included transient paresthesia (5.1%), postoperative infection requiring antibiotics (3.8%), wound dehiscence (2.6%) and persistent pain (2.6%). In the implant group, complications included peri-implantitis (3.8%), implant mobility (2.6%), temporary paresthesia (2.6%) and wound dehiscence (2.6%). Complication profiles are detailed in Table 3 (see PDF). Subgroup analysis revealed that posterior teeth demonstrated lower success rates compared to anterior teeth in Group A (85.1% vs. 96.8%, p = 0.089), while this difference was less pronounced in Group B (91.8% vs. 93.1%, p = 0.821). Lesion size greater than 10 mm was associated with reduced success rates in both groups, though statistical significance was reached only in Group A (p = 0.031). Age, sex and time since initial root canal treatment did not significantly influence outcomes in either group.

## Discussion:

The current research compared clinical outcomes between periapical surgery and extraction and replacement with implants in patients with chronic periapical pathology, showing that both treatment options yielded positive results, with no statistically significant difference in overall success rates at 24 months of follow-up. These results are consistent with modern knowledge that treatment choice should be personalized based on patient factors, rather than on the presumption that one drug is better than the other [16]. The periapical surgery group that is found to be successful (89.7) is consistent with what is already reported in the use of modern microsurgical procedures involving operating microscopes and biocompatible root-end filling materials [17]. With the introduction of mineral trioxide aggregate and bioceramic materials, periapical surgery has significantly improved due to the high sealing capacity and biocompatibility of these materials compared with traditional amalgam root-end fillings [18]. Modern micro-surgical techniques allow better visualization of anatomical complexity, which can also lead to treatment failure, such as isthmuses, lateral canals and micro-fractures that are difficult to see under traditional magnification [19]. This is an implant group with a high success rate of 92.3, within the range reported in systematic implant outcome studies for the extraction of teeth with periapical pathology [20]. The issue of possible contamination of the site of implantation by bacterial remnants of chronic periapical infection has been discussed in the literature, which indicates that effective debridement and appropriate healing guidelines reduce the risk of infection [21]. The current research incorporated immediate and delayed implant placement strategies and subgroup analysis showed no significant differences between them. That immediate implant placement can be performed in well-selected cases with the same level of safety. One critical result of this research was that the periapical surgery treatment period and the numbers of required appointments were much lower than those for implant placement. The average time spent in the surgical endodontic group was 2.1 months and 3.2 visits and the implant group took 7.8 months and 6.4 visits. Such disparity is indicative of the inherent complexity of implant therapy and therefore requires extraction healing, implant placement, bone integration and prosthetic fabrication [22]. Periapical surgery can be a more preferable choice over both treatments in cases where patients focus on the expedient treatment completion. Radiographic healing occurred more often in the implant group (87.2) than in the periapical surgery group (74.4) and this difference was statistically significant. Nevertheless, this observation must be taken with caution because partial healing in the periapical surgery group might still progress to total healing even after the 24-month evaluation period [23]. Past studies have reported that periapical healing after endodontic surgery can take a long time and some studies have shown persistent improvement up to 4 years of evaluation. The rate of complications was similar in both groups, with transient paresthesia being a more prevalent complication in the periapical surgery group and peri-implantitis being a complication peculiar to the implant group. In both groups, the occurrence of paresthesia was in the acceptable range and none of the occurrences of a permanent sensory disturbance was detected [24]. The case of 3.8% peri-implantitis is observed in the cases of implant therapy and highlights the necessity of proper maintenance procedures and adherence to the recall appointments by the patient. The financial aspect of treatment choice deserves to be considered in clinical decision-making. Although this study did not address a direct cost comparison as one of its main goals, the reduced number of appointments and shorter treatment period in periapical surgery would likely lead to lower total treatment costs [25]. On the other hand, implant restorations are subject to future prosthetic component replacement, which is a long-term maintenance expense not possible with retained natural teeth. There was no significant difference in patient satisfaction scores between the groups, both with mean scores above 8 on a 10-point scale. This fact implies that the choice of modality does not play a crucial role in how patients perceive their treatment success, as long as satisfactory functional and esthetic outcomes are achieved [26]. Education of patients about realistic expectations and the possible complications of each treatment option is a compulsory part of informed consent and the best possible satisfaction. Of significance is the impact of the positioning of teeth on the results of the treatment that was witnessed in this research. The success rate of the posterior teeth in the periapical surgery group was lower than that of the anterior teeth, probably due to the greater anatomical complexity and the reduced access of multirooted teeth [27]. The result justifies the use of extraction and placement of implants in the back teeth with complicated root structures when periapical surgery is expected to be more difficult or carry a higher risk of failure. The weaknesses of this study are that it is retrospective, which creates the possibility of selection bias in treatment allocation. The group of patients that underwent periapical surgery could have reported more positive prognostic factors that affected treatment choice. Also, the 24-month follow-up, which is sufficient for the preliminary outcome evaluation, might not capture delayed failures that may occur at longer follow-ups [28]. Future prospective randomized controlled trials with longer follow-ups would enhance the body of evidence on the comparative effectiveness of these treatment modalities. The results of this study endorse a tailored treatment-planning methodology that includes patient preferences, anatomy, restorative needs, economic (financial) considerations and perceived chances of success for each treatment option. Both periapical surgery and implant placement have no obvious advantages in all the possible outcome parameters, which seems to indicate that both interventions should be considered as first-line modalities of managing the persistent periapical pathology.

## Conclusion:

Periapical surgery and extraction-implant show ≥ 89% success/survival at 24 months for persistent periapical pathology, with no significant differences (p > 0.05). Surgery offers shorter treatment duration and fewer visits, while implants lead to radiographic healing. However, complication/satisfaction rates are comparable across groups. Thus, treatment choice should prioritize patient/tooth factors over modality, as both yield reliable outcomes without permanent adverse events.
